# Supplementary material for: Association between physical activity and changes in intestinal microbiota composition: A systematic review
Source: PLoS One. 2021 Feb 25;16(2):e0247039. doi: 10.1371/journal.pone.0247039 (PMC7906424; doi:10.1371/journal.pone.0247039)
Supplement: S1 Table — (DOCX) [file pone.0247039.s002.docx]

**S1 Table.** ROBINS-I risk of bias assessment summary: review authors' judgements about each methodological quality item for each included study in this review.

| **Study** | **Bias due to confounding** | **Bias in selection of participants into the study** | **Bias in classification/**  **measurement of intervention** | **Bias due to deviations from intended interventions** | **Bias because of missing data** | **Bias in measurement of outcomes** | **Bias in selection of the reported result** | **Overall** |
| --- | --- | --- | --- | --- | --- | --- | --- | --- |
| Clarke et al. 2014 | Moderate | Low | Low | N/A | Low | Low | Low | Low |
| Estaki et al. 2016 | Moderate | Low | Low | N/A | Low | Low | Low | Low |
| Bressa et al. 2017 | Moderate | Low | Low | N/A | Moderate | Low | Moderate | Moderate |
| Petersen et al. 2017 | Serious | Moderate | Moderate | N/A | Moderate | Moderate | Moderate | Moderate |
| Yang et al. 2017 | Moderate | Low | Low | N/A | Moderate | Moderate | Moderate | Moderate |
| Whisner et al. 2018 | Moderate | Moderate | Low | N/A | Moderate | Low | Moderate | Moderate |
| Durk et al. 2019 | Serious | Serious | Low | N/A | Moderate | Moderate | Serious | Serious |
| Jang et al. 2019 | Moderate | Low | Low | N/A | Low | Low | Low | Low |
| Allen et al. 2018 | Low | Moderate | Moderate | Moderate | Moderate | Low | Low | Moderate |
| Munuka et al. 2018 | Low | Moderate | Low | Moderate | Moderate | Moderate | Moderate | Moderate |
| Kern et al. 2020 | Low | Moderate | Low | Moderate | Low | Low | Low | Low |
| Zhao et al. 2018 | Low | Low | Low | N/A | Low | Low | Low | Low |
| Scheiman et al. 2019 | Moderate | Low | Low | N/A | Low | Low | Low | Low |
| Hampton-Marcell et al. 2020 | Serious | Low | Moderate | Moderate | Moderate | Moderate | Moderate | Moderate |
