# Supplementary material for: Association between physical activity and changes in intestinal microbiota composition: A systematic review
Source: PLoS One. 2021 Feb 25;16(2):e0247039. doi: 10.1371/journal.pone.0247039 (PMC7906424; doi:10.1371/journal.pone.0247039)
Supplement: Attachment — Submitted filename: Response to reviewers.docx [file pone.0247039.s003.docx]

January 14th, 2021

Bogotá D.C., Colombia

Jane Foster, PhD

Academic Editor

PLOS ONE

Dear Dr. Foster,

First of all, we want to thank your consideration with our manuscript. Please find below as requested the specific responses to the comments and suggestions raised by the reviewers.

**COMMENTS FROM REVIEWER # 1**

**COMMENT:** The topic of the manuscript is relevant and actual. In fact, the relationship between microbiota and physical activity could be the key factor between physical activity and neurodegenerative diseases, creating a "gut-muscle-brain" axis.

Recently it has been published an article:

- Ticinesi A, Mancabelli L, Tagliaferri S, Nouvenne A, Milani C, Del Rio D, Lauretani F, Maggio MG, Ventura M, Meschi T.
  The Gut-Muscle Axis in Older Subjects with Low Muscle Mass and Performance: A Proof of Concept Study Exploring Fecal Microbiota Composition and Function with Shotgun Metagenomics Sequencing. Int J Mol Sci. 2020 Nov 25;21(23):E8946. doi: 10.3390/ijms21238946

That could be used for explaining this relationship.

**RESPONSE:** We agree with the inclusion of this recent study. A brief reference to this article can be found in “discussion section” specifically between lines 319 to 329, reference 73.

**COMMENT:** And also this other article could be used for close this loop:

Ticinesi A, Lauretani F, Tana C, Nouvenne A, Ridolo E, Meschi T. Exercise and immune system as modulators of intestinal microbiome: implications for the gut-muscle axis hypothesis. Exerc Immunol Rev. 2019;25:84-95.

**RESPONSE:** We agree with the inclusion of this review. A brief reference to the article can be found in “discussion section” specifically between lines 384 to 395, reference 98.

**COMMENT**: and finally:

Ticinesi A, Tana C, Nouvenne A, Prati B, Lauretani F, Meschi T. Gut microbiota, cognitive frailty and dementia in older individuals: a systematic review. Clin Interv Aging. 2018 Aug 29;13:1497-1511.

**RESPONSE:** We agree with the inclusion of this review. A brief reference of the article can be found in “discussion section” specifically between lines 384 to 395, reference 97.

**COMMENTS FROM REVIEWER #2**

In this study, Aya et.al. present a systematic review of literature describing the association between physical activity and intestinal microbiota in human subjects. From the studies they analyzed, the authors have provided a comprehensive overview of how exercise affects intestinal microbiota in humans. The review is well written, succinct, and gives a good overview of the studies considered. However, I have the following comments:

**COMMENT:** There have been recent reviews on the topic of exercise and gut microbiome composition in athletes vs non-athletes. Almost all the reviews describe the changes of gut microbiome in terms of the composition. I urge the authors to include a table like the other tables that provides a description of the downstream functional changes associated with the compositional information. The discussion section provides a good template for this.

**RESPONSE**: We certainly agree with the inclusion of a new table named “Description of possible functional changes associate with gut microbiota in physical activity and sports activities” (Lines 284 to 289)

Additionally, some lines were included in the text to introduce and explain the table, these can be found in the lines: 266 – 267; 304 – 305; 379; 400.

**COMMENT:** There are minor typos in the document e.g. TD2 instead of T2D for Type II Diabetes.

**RESPONSE:** Typo mistakes were corrected in lines 64 and 74.
